# Hypertensive Disorders and Cardiovascular Severe Maternal Morbidity in the US, 2015-2019

**DOI:** 10.1001/jamanetworkopen.2024.36478

**Published:** 2024-10-03

**Authors:** Isabelle Malhamé, Kara Nerenberg, Kelsey McLaughlin, Sonia M. Grandi, Stella S. Daskalopoulou, Amy Metcalfe

**Affiliations:** 1 Department of Medicine, McGill University Health Centre, Montreal, Quebec, Canada; 2 Centre for Outcomes Research and Evaluation, Research Institute of the McGill University Health Centre, Montreal, Quebec, Canada; 3 Departments of Medicine, Obstetrics & Gynecology, and Community Health Sciences Cumming School of Medicine, University of Calgary, Calgary, Alberta, Canada; 4 Departments of Obstetrics and Gynecology, Sinai Health Systems and University of Toronto, Toronto, Ontario, Canada; 5 Department of Epidemiology, Dalla Lana School of Public Health, University of Toronto, Ontario, Canada; 6 Child Health Evaluative Sciences Program, The Hospital for Sick Children, Toronto, Ontario, Canada

## Abstract

**Importance:**

The incidence of severe maternal morbidity (SMM)—and particularly cardiovascular SMM (cvSMM), the first cause of pregnancy-related mortality—has been rising in North America. Although hypertensive disorders of pregnancy (HDP) are common, their association with cvSMM specifically remains unclear.

**Objective:**

To assess the association between individual subtypes of HDP and cvSMM, in addition to overall SMM, in a large, nationally representative sample.

**Design, Setting, and Participants:**

A population-based cohort study using the United States National Inpatient Sample was conducted. Individuals with obstetric deliveries between 2015 and 2019 were included. Data analysis was performed from October 2023 to February 2024.

**Exposure:**

HDP subtypes included gestational hypertension, chronic hypertension, preeclampsia without severe features, severe preeclampsia, and HELLP (hemolysis, elevated liver enzymes, and low platelet) syndrome.

**Main Outcomes and Measures:**

The primary outcome was a composite of cvSMM (including conditions such as pulmonary edema, stroke, and acute myocardial infarction) and the secondary outcome was a composite of overall SMM (including cvSMM and other conditions such as respiratory failure, severe postpartum hemorrhage, and sepsis). Adjusted risk ratios (aRRs) for the association between HDP subtypes and the outcomes were estimated using modified Poisson regression models adjusted for demographic and clinical characteristics.

**Results:**

Among 15 714 940 obstetric deliveries, 2 045 089 (13.02%) had HDP, 23 445 (0.15%) were affected by cvSMM, and 282 160 (1.80%) were affected by SMM. The mean (SD) age of the cohort was of 29 (6) years. The incidence of cvSMM was higher in participants with HDP than those without HDP (0.48% [9770 of 2 045 089] vs 0.10% [13 680 of 13 669 851]; *P* < .001). Participants with HELLP syndrome had the highest risk for cvSMM (aRR, 17.55 [95% CI, 14.67-21.01]), followed by severe preeclampsia (aRR, 9.11 [95% CI, 8.26-10.04]), and chronic hypertension (aRR, 3.57 [95% CI, 3.15-4.05]). Although HDP subtypes were also associated with overall SMM, the association with HELLP syndrome (aRR, 9.94 [95% CI, 9.44-10.45]), severe preeclampsia (aRR, 3.66 [95% CI, 3.55-3.78]), and chronic hypertension (aRR, 1.96 [95% CI, 1.88-2.03]) was attenuated compared with cvSMM.

**Conclusions and Relevance:**

In this cohort study, a graded relationship by severity characterized the association between HDP and cvSMM. Although all HDP subtypes were associated with an increased risk of overall SMM, the risk was more pronounced for cvSMM.

## Key Points

**Question:**

What is the association between subtypes of hypertensive disorders of pregnancy (HDP) and cardiovascular severe maternal morbidity during delivery hospitalization?

**Findings:**

In this population-based cohort study including more than 15 million deliveries, a graded relationship by severity characterized the association between HDP subtypes and cardiovascular severe maternal morbidity. Patients with HELLP (hemolysis, elevated liver enzymes, and low platelets) syndrome were at highest risk, followed by those with severe preeclampsia, and those with chronic hypertension.

**Meaning:**

These results suggest that all HDP subtypes, particularly HELLP syndrome, are important risk factors for cardiovascular severe maternal morbidity at delivery.

## Teaser

This cohort study investigates whether there is an association between individual subtypes of hypertensive disorders of pregnancy and cardiovascular severe maternal morbidity, in addition to overall SMM, in a large, nationally representative sample.

## Introduction

Severe maternal morbidity (SMM) refers to a set of unexpected adverse outcomes related to pregnancy, labor, childbirth, and the postpartum period resulting in severe illness, prolonged hospitalization, and/or long-term disability.^1,2^ The incidence of SMM has been increasing in the US, and for every person who dies during or after pregnancy, at least 100 more experience SMM.^3,4,5^ As a result, the World Health Organization (WHO) has recommended to focus efforts on SMM prevention to improve the quality of pregnancy care.^6^ It is important to recognize, however, that SMM, as a whole, includes a broad range of heterogeneous conditions potentially affecting multiple organ systems and that results from various pathophysiologic processes (eg, respiratory failure, severe postpartum hemorrhage, and sepsis). To this end, specific preventative strategies are challenging to develop and implement given the disparate and multisystemic nature of SMM.

Cardiovascular complications, affecting up to 8 per 10 000 delivery hospitalizations, have been rising in the US, and they have become the leading cause of pregnancy-related deaths in high-income settings.^7,8,9,10^ Importantly, most cardiovascular mortality events occur among patients without any known cardiovascular disease prior to conception.^7,10,11,12^ Thus, expanding the understanding of pregnancy-related cardiovascular morbidity (both causes and effective prevention) in the general obstetric population is urgently required to reduce the burden of SMM and improve maternal outcomes.

Cardiovascular SMM (cvSMM) includes the subset of severe cardiovascular morbidity (eg, heart failure, stroke, and acute myocardial infarction).^10,13,14,15^ Accordingly, cvSMM encompasses serious conditions that affect the cardiovascular system specifically, and that may result from common pathophysiologic pathways. Mitigation strategies designed for cvSMM reduction may include preemptive diuresis postpartum, personalized blood pressure targets, and early postpartum follow-up visits.^15^ Better characterization of cvSMM and its determinants would enable the development and implementation of preventative measures geared toward reducing cardiovascular morbidity and mortality among pregnant and postpartum women.

Hypertensive disorders of pregnancy (HDP), which affect at least 10% of pregnancies, have been increasing in North America and globally.^16,17,18,19^ While HDP predispose women to long-term cardiovascular morbidity,^20,21^ they are also associated with acute, short-term cardiovascular morbidity, as a third of individuals with cvSMM at delivery have a diagnosis of HDP.^10^ Moreover, while HDP, as a group of conditions, have been associated with SMM and mortality,^22,23^ the contribution of individual HDP subtypes (ie, chronic hypertension, gestational hypertension, preeclampsia without severe features, severe preeclampsia) to cvSMM and SMM remains understudied. Elucidating the association between HDP subtypes and cvSMM may lead to targeted resource allocation, thereby improving the quality of peripartum care. Thus, this study’s primary objective was to evaluate the association of HDP subtypes with cvSMM, in addition to overall SMM, in a nationally representative population-based sample in the US.

## Methods

This cohort study was exempt from institutional review board approval and did not require informed consent, as anonymized data from the National Inpatient Sample (NIS) are publicly available. To maintain confidentiality, cells from tables comprising 10 or fewer individuals using unweighted results were not reported. This report followed the Strengthening the Reporting of Observational Studies in Epidemiology (STROBE) reporting guideline.

### Data Sources and Study Population

We conducted a population-based cohort study using data from the NIS in the US. The NIS is an all-payer database that comprises data on a random sample of 20% of all hospital discharges that occur across the country.^24^ Deidentified data from hospital discharge abstracts are available for both patient-level demographics (eg, sex, age, race and ethnicity, median household income for zip code), diagnoses, and procedures.^24^ Information on race and ethnicity is provided by partner organizations using a unified variable, and reporting information can vary by hospital.^25^ Where both race and Hispanic ethnicity are supplied as separate elements, ethnicity takes precedence.^25^ The category *other* defines other state-reported categories excluded from NIS categories, and multiple racial categories.^26^ Information on race and ethnicity was reported to characterize racial and ethnic disparities in cvSMM in the context of HDP, and to account for confounding by interpersonal or structural forms of racism, which are known to be associated with both HDP and adverse perinatal outcomes.^26^

A validated definition was used to identify all obstetric deliveries between October 1, 2015, and December 31, 2019, in pregnant individuals between the ages of 12 and 55 years.^27^ The study period was chosen to ensure that data were coded consistently using *International Statistical Classification of Diseases, Tenth Revision, Clinical Modification (ICD-10-CM)* indicators,^28^ and that the hypertensive disorders of pregnancy were defined according to revised criteria by the American College of Obstetricians and Gynecologists adopted in 2013.^29^

### Definition of Exposure and Covariates

We used a hierarchical approach to define HDP exposure based on severity, as follows: gestational hypertension, chronic hypertension without preeclampsia, preeclampsia without severe features (comprising de novo preeclampsia and chronic hypertension with superimposed preeclampsia), severe preeclampsia, and HELLP (hemolysis, elevated liver enzymes, and low platelets) syndrome.^30^ Eclampsia was not considered as an exposure, as it was included in the outcome of SMM overall.^5^ HDP subtypes were considered as mutually exclusive, with the more severe being chosen in case of the occurrence of multiple HDP subtypes. See eTable 1 in Supplement 1 for codes.

We assessed demographic characteristics of the study population including insurance type, household income, race and ethnicity, maternal age, and day of admission (weekday vs weekend) both overall and by exposure to HDP. Clinical characteristics included cardiovascular conditions (ie, congenital heart disease, rheumatic heart disease, nonrheumatic valvular heart disease, chronic ischemic heart disease, diseases of the pulmonary circulation, aortic dilation, nonpregnancy-related cardiomyopathies, peripartum cardiomyopathy, and other cardiac diseases), and other chronic conditions, such as asthma, chronic kidney disease, preexisting (type 1 and type 2) and gestational diabetes, hypo- and hyperthyroidism, obesity, obstructive sleep apnea, systemic lupus erythematosus, and illicit drug or tobacco use. Multiple gestation and mode of delivery were also described. See eTable 2 in Supplement 1 for codes used.

### Definition of Outcomes

The primary outcome of cvSMM was identified using a previously validated algorithm to group cardiovascular types of SMM conditions, as defined by the Centers for Disease Control and Prevention (CDC).^13,31^ This primary composite outcome of cvSMM included any one of acute myocardial infarction, aneurysm, cardiac arrest and/or ventricular fibrillation, conversion of cardiac rhythm, heart failure and/or arrest during surgery or procedure, puerperal cerebrovascular disorders, pulmonary edema and/or acute heart failure, and shock.^13,15^ This composite measure of cvSMM has previously been found to have comparable validity to the larger CDC algorithm for SMM.^13,32^

The secondary outcome was defined as a composite of all SMM using the larger CDC algorithm (ie, comprising any one of acute kidney failure, adult respiratory distress syndrome, amniotic fluid embolism, disseminated intravascular coagulation, eclampsia, severe anesthesia complications, sepsis, sickle cell disease with crisis, air and thrombotic embolism, blood products transfusion, hysterectomy, temporary tracheostomy, and ventilation in addition to cvSMM).^31^ Refer to eTable 3 in Supplement 1 for a complete list of *ICD-10-CM* codes.

### Statistical Analysis

Baseline demographic and clinical characteristics of participants with and without HDP were compared using χ^2^ test and *t* test for categorical and continuous variables, respectively. Crude and adjusted risk ratios (RRs) were estimated using modified Poisson regression models for the association between HDP subtypes and the primary and secondary outcomes. Separate models were fit for both cvSMM and overall SMM with HDP subtypes as the primary exposure. Models were adjusted for the demographic and clinical characteristics (as aforementioned) with known associations with both HDP subtypes and the clinical outcomes. Moreover, to address the known association between morbidity and mortality among women with cardiac comorbidity and concomitant HDP,^33^ models also included an interaction term between cardiac comorbidity and HDP subtype. Models were weighted to ensure estimates were nationally representative. Statistical significance was set at 2-sided *P* <.05. All analyses were performed using Stata SE version 14 (StataCorp) from October 2023 to February 2024.

## Results

**Figure 1.**
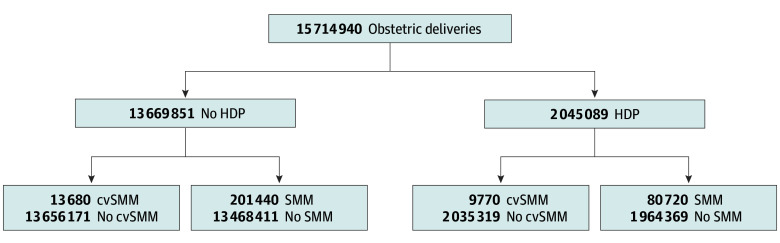
Flow Diagram of the Study Population Abbreviations: cvSMM, cardiovascular severe maternal morbidity; HDP, hypertensive disorders of pregnancy; SMM, severe maternal morbidity.

Among 15 714 940 obstetric deliveries during the study period, 2 045 089 (13.02%) had HDP (Figure 1). Mean (SD) age of the cohort was of 29 (6) years (Table 1). Hypertensive disorders were more common among persons with household income less than $39 000 (31.22% [632 630 of 2 045 089] vs 27.70% [3 749 548 of 13 669 851]; *P* < .001), Black individuals (20.24% [413 880 of 2 045 089] vs 13.48% [1 842 785 of 13 669 851]; *P* < .001), and participants at least 40 years of age (6.17% [126 170 of 2 045,089] vs 3.37% [460 660 of 13 669 851]; *P* < .001) (Table 1). Cardiac comorbidities were more prevalent in participants with HDP, including chronic ischemic heart disease (0.19% [3880 of 2 045 089] vs 0.05% [6595 of 13 669 851]; *P* < .001) and cardiomyopathy (0.12% [2450 of 2 045 089] vs 0.03% [4330 of 1 3669 851]; *P* < .001) (Table 2). The prevalence of cardiometabolic conditions, such as prepregnancy diabetes (3.6% [73 525 of 2 045 089] vs 0.76% [103 310 of 13 669 851]; *P* < .001), gestational diabetes (11.82% [241 650 of 2 045 089] vs 6.99% [956 084 of 13 669 851]; *P* < .001), and obesity (23.55% [481 729 of 2 045 089] vs. 8.79% [1 201 360 of 13 669 851]; *P* < .001) was also higher among persons with HDP (Table 2). More patients with HDP delivered via cesarean birth than those without HDP (43.63% [892 360 of 2 045 089] vs 30.2% [4 128 552 of 13 669 851]; *P* < .001) (Table 2).

**Table 1.** Baseline Demographic Characteristics of the Study Population

Characteristics	Participants, No. (%)			
	Total (n = 15 714 940)	No HDP (n = 13 669 851)	Any HDP (n = 2 045 089)	*P* value^a^
Insurance type				
Public insurance	6 799 086 (43.32)	5 913 631 (43.31)	885 455 (43.35)	<.001
Private insurance	8 048 679 (51.28)	6 982 415 (51.14)	1 066 265 (52.20)	<.001
Other	848 274 (5.40)	757 430 (5.55)	90 845 (4.45)	<.001
Household income, $				
<39 999	4 382 177 (28.15)	3 749 548 (27.70)	632 630 (31.22)	<.001
40 000-50 999	3 909 842 (25.12)	3 396 982 (25.09)	512 860 (25.31)	<.001
51 000-65 999	3 860 142 (24.8)	3 364 433 (24.85)	495 710 (24.46)	<.001
≥66 000	3 412 313 (21.92)	3 026 928 (22.36)	385 385 (19.02)	<.001
Race and ethnicity				
Asian or Pacific Islander	932 919 (5.94)	856 599 (6.27)	76 320 (3.73)	<.001
Black	2 256 665 (14.36)	1 842 785 (13.48)	413 880 (20.24)	<.001
Hispanic	3 117 298 (19.84)	2 771 998 (20.28)	345 300 (16.88)	<.001
Native American	110 930 (0.71)	95 085 (0.70)	15 845 (0.77)	<.001
Other^b^	691 930 (4.40)	618 460 (4.52)	73 470 (3.59)	<.001
Unknown	694 174 (4.42)	615 269 (4.50)	78 905 (3.86)	<.001
White	7 911 024 (50.34)	6 869 655 (50.25)	1 041 369 (50.92)	<.001
Age, y				
Mean (SD)	29 (6)	28.9 (5.9)	29.6 (6.5)	<.001
<20	808 374 (5.14)	702 160 (5.14)	106 215 (5.19)	<.001
20-24	3 073 218 (19.56)	2 701 558 (19.76)	371 660 (18.17)	<.001
25-29	4 525 307 (28.80)	3 983 827 (29.14)	541 480 (26.48)	<.001
30-34	4 444 417 (28.28)	3 885 708 (28.43)	558 710 (27.32)	<.001
35-39	2 276 794 (14.49)	1 935 939 (14.16)	340 855 (16.67)	<.001
≥40	586 830 (3.73)	460 660 (3.37)	126 170 (6.17)	<.001
Day of admission				
Weekday	12 605 267 (80.21)	10 881 917 (79.61)	1 723 349 (84.27)	<.001
Weekend	3 109 663 (19.79)	2 787 923 (20.39)	321 740 (15.73)	<.001

Abbreviation: HDP, hypertensive disorders of pregnancy.

^a^ χ^2^ test and *t* test were used to compare proportions and mean (SD) between participants with any HDP and without HDP, respectively.

^b^ As defined by Healthcare Cost & Utilization Project Partner organizations.^25^

**Table 2.** Baseline Clinical Characteristics of the Study Population

Characteristics	Participants, No. (%)			
	Total (n = 15 714 940)	No HDP (n = 13 669 851)	Any HDP (n = 2 045 089)	*P* value^a^
Hypertensive disorders of pregnancy				
Gestational hypertension	804 885 (5.12)	NA	NA	NA
Chronic hypertension	376 595 (2.40)	NA	NA	NA
Preeclampsia without severe features	480 305 (3.06)	NA	NA	NA
Severe preeclampsia	340 045 (2.16)	NA	NA	NA
HELLP syndrome	43 260 (0.28)	NA	NA	NA
Cardiac conditions^b^	62 940 (0.40)	45 715 (0.33)	17 225 (.84)	<.001
Congenital heart disease	16 575 (0.11)	13 335 (0.10)	3240 (.16)	<.001
Valvular heart disease	26 320 (0.17)	20 525 (0.15)	5795 (.28)	<.001
Chronic rheumatic heart disease	5185 (0.03)	3745 (0.03)	1440 (.07)	<.001
Chronic ischemic heart disease	10 475 (0.07)	6595 (0.05)	3880 (.19)	<.001
Diseases of pulmonary circulation	5315 (0.03)	3320 (0.02)	1995 (.10)	<.001
Aortic dilation	655 (<0.01)	495 (<0.01)	160 (<.01)	<.001
Cardiomyopathy^c^	6780 (0.04)	4330 (0.03)	2450 (.12)	<.001
Peripartum cardiomyopathy	3285 (0.02)	1530 (0.01)	1755 (.09)	<.001
Asthma	791 235 (5.03)	639 625 (4.68)	151 610 (7.41)	<.001
Chronic kidney disease	28 140 (0.18)	19 540 (0.14)	8600 (.42)	<.001
Type 1 and 2 diabetes	176 835 (1.13)	103 310 (0.76)	73 525 (3.60)	<.001
Gestational diabetes	1 197 734 (7.62)	956 084 (6.99)	241 650 (11.82)	<.001
Hypothyroidism	579 550 (3.69)	476 695 (3.49)	102 855 (5.03)	<.001
Hyperthyroidism	39 740 (0.25)	31.600 (0.23)	8140 (.40)	<.001
Obesity	1 683 079 (10.71)	1 201 360 (8.79)	481 729 (23.55)	<.001
Obstructive sleep apnea	31 215 (0.20)	17 175 (0.13)	14 040 (.69)	<.001
Systemic lupus erythematosus	24 730 (0.16)	18 495 (0.14)	6235 (.30)	<.001
Illicit drug or tobacco use	419 055 (2.67)	349 950 (2.56)	69 105 (3.38)	<.001
Multiple gestation	279 725 (1.78)	208 480 (1.53)	71 245 (3.48)	<.001
Delivery mode				
Spontaneous vaginal delivery	10 046 123 (63.93)	8 968 929 (65.61)	1 077 194 (52.67)	<.001
Operative vaginal delivery	647 905 (4.12)	572 370 (4.19)	75 535 (3.69)	<.001
Cesarean delivery	5 020 912 (31.95)	4 128 552 (30.20)	892 360 (43.63)	<.001

Abbreviations: HDP, hypertensive disorders of pregnancy; HELLP, hemolysis, elevated liver enzymes, and low platelets; NA, not applicable.

^a^ χ^2^ test was used to compare proportions between participants with any HDP and without HDP.

^b^ Categories were not mutually exclusive.

^c^ Except peripartum cardiomyopathy.

**Figure 2.**
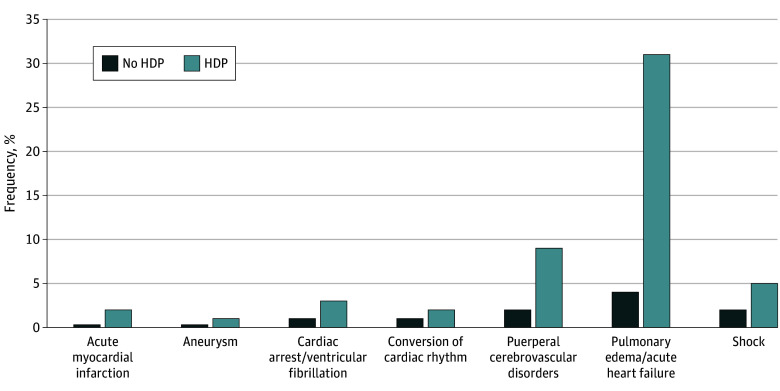
Distribution of Cardiovascular Severe Maternal Morbidity Among Individuals With and Without HDP^a^ HDP indicates hypertensive disorders of pregnancy. ^a^All differences were statistically significant with *P* < .001.

Overall, 23 445 deliveries (0.15%) were complicated by cvSMM and 282 160 (1.8%) were complicated by overall SMM (Table 3). Pulmonary edema and/or acute heart failure and cerebrovascular disorders were the most common cvSMM events (Figure 2; eTable 4 in Supplement 1). The incidence of cvSMM was approximately 5 times higher in patients with HDP than those without HDP (0.48% [9770 of 2 045 089] vs 0.10% [13 680 of 13 669 851]; *P* < .001) (eTable 4 in Supplement 1). The proportion of patients with acute myocardial infarction (0.02% [340 of 2 045 089] vs 0.003% [400 of 13 669 851]; *P* < .001), aneurysm (0.01% [210 of 2 045 089] vs 0.003% [465 of 13 669 851]; *P* < .001), cardiac arrest and/or ventricular fibrillation (0.03% [545 of 2 045 089] vs 0.01% [1 235 of 13 669 851]; *P* < .001), conversion of cardiac rhythm (0.02% [455 of 2 045 089] vs 0.01% [1090 of 13 669 851]; *P* < .001), puerperal cerebrovascular disorders (0.09% [1740 of 2 045 089] vs 0.02% [2775 of 13 669 851]; *P* < .001), pulmonary edema and/or acute heart failure (0.31% [6244 of 2 045 089] vs 0.04% [5935 of 13 669 851]; *P* < .001), and shock (0.05% [1060 of 2 045 089] vs 0.02% [3310 of 13 669 851]; *P* < .001) was significantly higher among individuals with HDP compared with individuals without HDP (Figure 2; eTable 4 in Supplement 1).

**Table 3.** Association Between Hypertensive Disorders of Pregnancy Subtypes and Severe Maternal Morbidity (Cardiovascular and Overall)

Variable	cvSMM (n = 23 445)		SMM (n = 282 160)	
	Unadjusted RR (95% CI)	Adjusted RR (95% CI)^a^	Unadjusted RR (95% CI)	Adjusted RR (95% CI)^a^
Gestational hypertension	1.15 (1.00-1.34)	1.19 (1.00-1.40)	1.34 (1.30-1.39)	1.33 (1.29-1.38)
Chronic hypertension	6.42 (5.83-7.07)	3.57 (3.15-4.05)	2.92 (2.82-3.03)	1.96 (1.88-2.03)
Preeclampsia without severe features	3.81 (3.41-4.25)	2.86 (2.49-3.27)	2.57 (2.48-2.66)	2.17 (2.10-2.25)
Severe preeclampsia	11.23 (10.36-12.16)	9.11 (8.26-10.04)	4.61 (4.48-4.75)	3.66 (3.55-3.78)
HELLP syndrome	17.79 (15.14-20.89)	17.55 (14.67-21.01)	11.38 (10.85-11.94)	9.94 (9.44-10.45)

Abbreviations: cvSMM, cardiovascular severe maternal morbidity; HELLP, hemolysis, elevated liver enzymes, and low platelets; SMM, severe maternal morbidity; RR, risk ratio.

^a^ Adjusted for type of insurance, income, race and ethnicity, day of admission, age, cardiac comorbidity, asthma, chronic kidney disease, pre-existing and gestational diabetes, hypothyroidism, hyperthyroidism, obesity, obstructive sleep apnea, systemic lupus erythematosus, illicit drug or tobacco use, multiple gestation pregnancy, and an interaction term between cardiac comorbidity and HDP subtype.

While all HDP subtypes were associated with cvSMM during delivery hospitalization, there was a gradient based on severity with HELLP syndrome having the highest risk (adjusted RR, 17.55 [95% CI, 14.67-21.01]), followed by severe preeclampsia (adjusted RR, 9.11 [95% CI, 8.26-10.04]), and chronic hypertension (adjusted RR, 3.57 [95% CI, 3.15-4.05]) (Table 3). Although HELLP syndrome remained an important risk factor for SMM overall (adjusted RR, 9.94 [95% CI, 9.44-10.45]), its association with SMM was less pronounced than with cvSMM specifically (Table 3). Similarly, the association with SMM was attenuated for severe preeclampsia (adjusted RR, 3.66 [95% CI, 3.55-3.78]) and chronic hypertension (adjusted RR, 1.96 [95% CI, 1.88-2.03]) (Table 3). Gestational hypertension was found to be associated with both cvSMM (adjusted RR, 1.19 [95% CI, 1.00-1.40]) and SMM (adjusted RR, 1.33 [95% CI, 1.29-1.38]), although to a lesser extent than other HDP subtypes (Table 3).

## Discussion

We conducted a population-based cohort study using data from the NIS in the US to evaluate the association between HDP subtypes and cvSMM during hospitalization for delivery. The frequency of HDP was higher among participants with low household income, Black individuals, and persons at least 40 years of age. Pulmonary edema and stroke were the most common cvSMM events to occur. Overall, the proportion of participants with cvSMM was higher among individuals with HDP compared with those without HDP. All HDP subtypes were associated with cvSMM, with a graded trend based on severity of HDP subtype. The strength of the association between HDP and cvSMM was highest among individuals with HELLP syndrome and lowest among individuals with gestational hypertension. While all HDP subtypes were also associated with SMM, the association with chronic hypertension, preeclampsia without severe features, severe preeclampsia, and HELLP syndrome was attenuated compared with cvSMM.

Pulmonary edema and/or acute heart failure events and strokes were the most common cvSMM events among patients with HDP at delivery hospitalization. Pulmonary edema and acute heart failure in the context of HDP may result from several pathophysiologic mechanisms. First, preeclampsia is associated with patterns of cardiac remodeling manifesting clinically as heart failure with preserved or mildly reduced ejection fraction.^34^ In addition, preeclampsia is diagnosed in approximately 20% of patients with peripartum cardiomyopathy, a pregnancy-specific cause of heart failure with reduced ejection fraction.^34,35,36^ Moreover, increased afterload from severe hypertension and low oncotic pressure from proteinuria may contribute to pulmonary edema in the context of HDP.^36,37^ Similarly, pregnancy-associated stroke may result from several entities closely linked with HDP, including eclampsia, posterior reversible encephalopathy syndrome (PRES), and reversible cerebral vasoconstriction syndrome.^38,39,40^ As pulmonary edema is present in approximately 1 in 10 patients who die from cardiovascular complications at delivery,^10^ and stroke is the leading cause of pregnancy-related long-term disability,^41^ our findings suggest that HDP remain substantial contributors to maternal morbidity and mortality, despite advances in obstetric care.

Our study found a 17.6-fold increased risk of developing cvSMM in the context of HELLP syndrome. Although an association between HDP and cvSMM has previously been identified,^42^ we herein describe a graded relationship with HDP subtype severity, which highlights the prominent association of HELLP syndrome with cvSMM. This observed association may be explained by the presence of microvascular dysfunction in both HELLP syndrome and cvSMM. Indeed, dysfunction in the hepatic microcirculation, initiated by vasoactive factors and inflammatory cytokines, is a proposed mechanism for liver injury characterizing HELLP syndrome and coronary microvascular alterations in patients with preeclampsia.^43,44,45^ Moreover, magnetic resonance imaging findings consistent with microvascular endothelial dysfunction are observed in the context of HDP in women with preeclampsia and neurologic manifestations (including eclampsia and PRES), which may precede the development of pregnancy-associated stroke.^44,46,47,48,49^ Our findings support the concept that individuals who go on to develop cvSMM in the context of HDP, and particularly HELLP syndrome, may represent a subset of patients with more pronounced systemic vascular dysfunction in the acute phase of HDP, potentially underlying the lifelong increased risk of cardiovascular disease after HDP.

This investigation found that the association between all HDP subtypes (except gestational hypertension) and cvSMM was more pronounced than their association with overall SMM. This may in part be explained by the fact that SMM as a composite outcome includes a heterogeneous set of conditions representing dysfunction from multiple organ systems, and that this outcome measure may be less specific to HDP. Understanding the risk of SMM in general through the use of risk assessment tools^22,50,51^ is a promising means to identify individuals who may benefit from delivery at designated health centers with higher levels of maternal care.^52,53^ However, improving the characterization of cvSMM risk could lead to more specific mitigation strategies. Accordingly, our findings delineate a differential risk of cvSMM according to HDP subtype, which supports the need to develop and evaluate cardiovascular interventions tailored to HDP subtypes during the acute setting. Patients with HDP subtypes at higher risk for cvSMM may benefit from proactive preventative strategies, including: intensive blood pressure treatment to achieve blood pressure targets and prevent severe hypertension, inpatient monitoring protocols during the early postpartum period, enhanced outpatient monitoring of blood pressure and clinical symptoms, and comprehensive and regular postpartum cardiovascular health assessments. Furthermore, reducing the burden of acute cvSMM may be an important strategy to improve the long-term cardiovascular outcomes of persons with HDP.

Our study had several strengths. The recent study period allowed for a contemporaneous overview of the relationship between HDP and cvSMM, addressing an important and unmet clinical need. Moreover, the use of *ICD-10-CM* coding enabled us to distinguish HELLP syndrome from other HDP subtypes, thereby highlighting its pivotal contribution to adverse cardiovascular outcomes at delivery. Using a large, representative sample, we identified novel significant associations between HDP and relatively rare outcomes among a diverse patient population, increasing the generalizability of our findings.

### Limitations

Our study had limitations. First, this administrative dataset did not include detailed clinical information which may be associated with cvSMM and overall SMM, such as body mass index, the presence of cardiovascular signs and symptoms, and echocardiographic and other imaging or laboratory results. Moreover, the dataset did not allow for linkage of consecutive hospitalizations per patient. As such, recurrent deliveries could not be assessed, and postpartum readmissions could not be evaluated. In addition, the extent to which misclassification bias of exposures and outcomes may have affected our results is not known. For instance, cases of eclampsia, an outcome measure associated with HDP by definition, may have been misclassified as unexposed to HDP. Moreover, as definitions for severe preeclampsia may include cvSMM outcomes (eg, pulmonary edema), some cvSMM events may be underreported.^13^ As a result, the associations observed in our study may have been attenuated. Although the association between HDP at delivery and postpartum adverse cardiovascular outcomes has been examined,^10,15^ additional research evaluating this association in the postpartum period at a national scale is required. Additionally, future work to better characterize the complex relationship between cardiometabolic risk factors (including obesity and weight gain across the pregnancy continuum), severity of HDP phenotypes, and cvSMM, would be needed.

## Conclusions

This cohort study found that all HDP subtypes were associated with cvSMM events, mostly driven by the increased incidence of pulmonary edema and stroke. There was a graded trend in this association based on severity of HDP subtype, with HELLP syndrome exhibiting the strongest association with cardiovascular morbidity. While all HDP subtypes were associated with an increased risk of SMM overall, the risk was stronger for cvSMM specifically. Together, these findings highlight the urgent need for improved peripartum cardiovascular care in the short-term, in addition to efforts focused on long-term cardiovascular risk reduction. Further research is required to identify mechanisms and therapeutic targets linking HELLP syndrome and acute cardiovascular morbidity in the context of HDP.

## Notes

Supplement 1. **eTable 1.** ICD-10 Codes Used to Identify Hypertensive Disorders of Pregnancy **eTable 2.** ICD-10 Codes Used to Identify Clinical Characteristics **eTable 3.** Standard ICD-10 Codes Used to Identify Severe Maternal Morbidity (Cardiovascular and Overall) **eTable 4.** Frequency of Cardiovascular Severe Maternal Morbidity Conditions Among Women With and Without Hypertensive Disorders of Pregnancy

Supplement 2.
Data Sharing Statement
