# Supplementary material for: Hypertensive Disorders and Cardiovascular Severe Maternal Morbidity in the US, 2015-2019
Source: JAMA Netw Open. 2024 Oct 3;7(10):e2436478. doi: 10.1001/jamanetworkopen.2024.36478 (PMC11581633; doi:10.1001/jamanetworkopen.2024.36478)
Supplement: Supplement 1. — eTable 1. ICD-10 Codes Used to Identify Hypertensive Disorders of Pregnancy eTable 2. ICD-10 Codes Used to Identify Clinical Characteristics eTable 3. Standard ICD-10 Codes Used to Identify Severe Maternal Morbidity (Cardiovascular and Overall) eTable 4. Frequency of Cardiovascular Severe Maternal Morbidity Conditions Among Women With and Without Hypertensive Disorders of Pregnancy [file jamanetwopen-e2436478-s001.pdf]

Supplemental Online Content

Malhamé I, Nerenberg K, McLaughlin K, Grandi SM, Daskalopoulou SS, Metcalfe A. Hypertensive disorders and cardiovascular severe maternal morbidity in the US, 2015-2019. *JAMA Netw Open.* 2024;7(9):e2436478.
doi:10.1001/jamanetworkopen.2024.36478

eTable 1. ICD-10 Codes Used to Identify Hypertensive Disorders of Pregnancy

eTable 2. ICD-10 Codes Used to Identify Clinical Characteristics

eTable 3. Standard ICD-10 Codes Used to Identify Severe Maternal Morbidity (Cardiovascular and Overall)

eTable 4. Frequency of Cardiovascular Severe Maternal Morbidity Conditions Among Women With and Without Hypertensive Disorders of Pregnancy

This supplemental material has been provided by the authors to give readers additional information about their work.

© 2024 Malhamé I et al. *JAMA Network Open.*

eTable 1. ICD-10 codes used to identify hypertensive disorders of pregnancy

Condition	Codes
Gestational hypertension	O13
Chronic hypertension without preeclampsia	O10, I10
Preeclampsia without severe features	O14, O11
Severe preeclampsia	O14.1
HELLP syndrome	O12.2

HELLP = Hemolysis, Elevated Liver enzymes, and Low platelets

eTable 2. ICD-10 codes used to identify clinical characteristics

Condition	Codes
Cardiac comorbidities	
Congenital heart disease	CHD – lesion, severe: Q20.0, Q20.1, Q20.3, Q20.4, Q20.5. Q20.8, Q21.2, Q21.3, Q22.5, Q23.4<br>CHD lesion – shunt Q21.0, Q21.1, Q21.9, I27.83, Q25.0<br>CHD – coarctation Q25.1, Q25.21, Q25.29<br>CHD – valve Q22.0, Q22.1, Q22.2, Q22.3, Q22.9, Q23.0, Q23.1, Q23,2, Q23.3, Q25.5, Q25.6, Q25.71, Q25.72, Q25.79<br>CHD – other Q24.0, Q24.1, Q24.2, Q24.3, Q24.4, Q24.5, Q24.6, Q24.8, Q23.8, Q20.9, Q24.9, Q25.40, Q25.41, Q25.42, Q25.43, Q25.44, Q25.45, Q25.46, Q25.47, Q25.48, Q25.49, Q25.29, Q25.3, Q25.60, Q25.61, Q25.62, Q25.63, Q25.68, Q25.69, Q28.9
Valvular heart disease	I34.x, I35.x, I36.x, I37.x, I38, I39
Chronic rheumatic heart disease	I05.x, I06.x, I07.x, I08.x, I09.x
Chronic ischemic heart disease	I25.x
Diseases of pulmonary circulation	I27.x, I28.x
Aortic dilation	I77.81x
Cardiomyopathy	I42.x, I43x
Peripartum cardiomyopathy	O90.3
Asthma	J45.x
Chronic kidney disease	N18.x, N19.x, T82.4, Z49.2, Z99.2
Type 1 and 2 diabetes	E10.x, E11.x, E13.x, E14.x, O24.8
Gestational diabetes	O24.4xx
Hypothyroidism	E02.x, E03.x, E06.x
Hyperthyroidism	E05.x
Illicit drug or tobacco use	F10-F19, F55, G31.2, O35.4, O35.5, T51.x, T65.2, Z72.0, Z72.1, Z72.2
Multiple gestation	O30.0-O30.2, O30.8-O30.9, O31, Z37.2-Z37.7, Z37.90
Obesity	E66.x, O99.21
Obstructive sleep apnea	G47.3
Systemic lupus erythematosus	M32
Delivery mode	
Spontaneous vaginal delivery	O80.x
Operative vaginal delivery	10D07Z3, 10D07Z4, 10D07Z5, 10D07Z6, 10D07Z7, 10D07Z8
Caesarean section delivery	10D00Z0, 10D00Z1, 10D00Z2

CHD = Congenital heart disease

© 2024 Malhamé I et al. *JAMA Network Open.*

eTable 3. Standard ICD-10 codes used to identify severe maternal morbidity (cardiovascular and overall)[1]

Condition	Codes
**Cardiovascular SMM**	
Acute myocardial infarction	I21.01, I21.02, I21.09, I21.11, I21.19, I21.21, I21.29, I21.3, I21.4, I21.9, I21.A1, I21.A9, I22.0, I22.1, I22.2, I22.8, I22.9
Aneurysm	I71.00, I71.01, I71.02, I71.03, I71.1, I71.2, I71.3, I71.4, I71.5, I71.6, I71.8, I71.9, I79.0
Cardiac arrest/ventricular fibrillation	I46.2, I46.8, I46.9, I49.01, I49.02
Heart failure/arrest during surgery or procedure	I97.120, I97.121, I97.130, I97.131, I97.710, I97.711
Puerperal cerebrovascular disorders	I60.0, I60.1, I60.2, I60.3, I60.4, I60.5, I60.6, I60.7, I60.8, I60.9, I61.1, I61.2, I61.3, I61.4, I61.5, I61.6, I61.8, I61.9, I62.0, I62.1, I62.9, I63.0, I63.1, I63.2, I63.3, I63.4, I63.5, I63.6, I63.8, I63.9, I65.0, I65.1, I65,2, I65.8, I65.9, I66.0, I66.1, I66.2, I66.3, I66.8, I66.9, I67.0, I67.1, I67.2, I67.3, I67.4, I67.5, I67.6, I67.7, I67.8, I67.9, I68.0, I68.2, I68.8O22.51, O22.52, O22.53, I97.810, I97.811, I97.820, I97.821, O87.3
Pulmonary edema/acute heart failure	J81.0, I50.1, I50.20, I50.21, I50.23, I50.30, I50.31, I50.33, I50.40, I50.41, I50.43, I50.9
Conversion of cardiac rhythm	5A2204Z, 5A12012
Cardiogenic Shock	R57, T81.10XA, T81.11XA, T81.19XA
**SMM**	
Acute myocardial infarction	I21.01, I21.02, I21.09, I21.11, I21.19, I21.21, I21.29, I21.3, I21.4, I21.9, I21.A1, I21.A9, I22.0, I22.1, I22.2, I22.8, I22.9
Aneurysm	I71.00, I71.01, I71.02, I71.03, I71.1, I71.2, I71.3, I71.4, I71.5, I71.6, I71.8, I71.9, I79.0
Acute renal failure	N17.0, N17.1, N17.2, N17.8, N17.9, O90.4
Adult respiratory distress syndrome	J80, J95.1, J95.2, J95.3, J95.821, J95.822, J96.00, J96.01, J96.02, J96.20, J96.21, J96.22, R09.2
Amniotic fluid embolism	O88.11, O88.12, O88.13
Cardiac arrest/ventricular fibrillation	I46.2, I46.8, I46.9, I49.01, I49.02
Conversion of cardiac rhythm	5A2204Z, 5A12012
Disseminated intravascular coagulation	D65, D68.8, D68.9, O72.3
Eclampsia	O15.00, O15.02, O15.03, O15.1, O15.2, O15.9
Heart failure/arrest during surgery or procedure	I97.120, I97.121, I97.130, I97.131, I97.710, I97.711
Puerperal cerebrovascular disorders	I60.0, I60.1, I60.2, I60.3, I60.4, I60.5, I60.6, I60.7, I60.8, I60.9, I61.1, I61.2, I61.3, I61.4, I61.5, I61.6, I61.8, I61.9, I62.0, I62.1, I62.9, I63.0, I63.1, I63.2, I63.3, I63.4, I63.5, I63.6, I63.8, I63.9, I65.0, I65.1, I65,2, I65.8, I65.9, I66.0, I66.1, I66.2, I66.3, I66.8, I66.9, I67.0, I67.1, I67.2, I67.3, I67.4, I67.5, I67.6, I67.7, I67.8, I67.9, I68.0, I68.2, I68.8O22.51, O22.52, O22.53, I97.810, I97.811, I97.820, I97.821, O87.3

Pulmonary edema/acute heart failure	J81.0, I50.1, I50.20, I50.21, I50.23, I50.30, I50.31, I50.33, I50.40, I50.41, I50.43, I50.9
Severe anesthesia complications	O74.0, O74.1, O74.2, O74.3, O80.01, O89.09, O89.1, O89.2
Sepsis	A40.0, A40.1, A40.3, A40.8, A40.9, A41.01, A41.02, A41.1, A41.2, A41.3, A41.50, A41.51, A41.52, A41.53, A41.59, A41.81, A41.89, A41.9, A32.7, O85, T80.211A, T81.4XXA, T81.44, T81.44XA, T81.44XD, T81.44XS, R65.20
Shock	O75.1, R57.0, R57.1, R57.8, 557.9, R65.21, T78.2XXA, T88.2XXA, T88.6XXA, T81.10XA, T81.11XA, T81.19XA
Sickle cell disease with crisis	D57.00, D57.01, D57.02, D57.211, D57.212, D57.219, D57.411, D57.412, D57.419, D57.811, D57.812, D57.819
Air and thrombotic embolism	O88.011, O88.012, O88.013, O88.014, O88.015, O88.016, O00.017, O88.018, O88.019, O88.02, O88.03, O88.211, O88.212, O88.213, O88.214, O88.215, O88.216, O88.217, O88.218, O88.219, O88.22. O88.23, O88.311, O88.312, O88.313, O88.314, O88.315, O88.316, O88.317, O88.318, O88.319, O88.32, O88.33, O88.81, O88.82, O88.83, I26.01, I26.02, I26.09, I26.90, I26.92, I26.99
Blood products transfusion	30233H1, 30233K1, 30233L1, 30233M1, 30233N1, 30233P1, 30233R1, 30233T1, 30240H1, 30240K1, 30240L1, 30240M1, 30240N1, 30240P1, 30240R1, 30240T1, 30243H1, 30243K1, 30243L1, 30243M1, 30243N1, 30243P1, 30243R1, 30243T1
Hysterectomy	0UT90ZZ, 0UT94ZZ, 0UT97ZZ, 0UT98ZZ, 0UT9FZZ
Temporary tracheostomy	0B110Z4, 0B110F4, 0B113Z4, 0B113F4, 0B114Z4, 0B114F4
Ventilation	5A1935Z, 5A1945Z, 5A1955Z

SMM = Severe Maternal Morbidity

© 2024 Malhamé I et al. *JAMA Network Open.*

eTable 4. Frequency of cardiovascular severe maternal morbidity conditions among women with and without hypertensive disorders of pregnancy

Condition	Total N= 15,714,940	No HDP N=13,669,851	Any HDP N=2,045,089
Any cardiovascular severe maternal morbidity	23,445 (0.15)	13,680 (0.10)	9,770 (0.48)
Acute myocardial infarction	740 (0.00)	400 (0.00)	340 (0.02)
Aneurysm	675 (0.00)	465 (0.00)	210 (0.01)
Cardiac arrest/ventricular fibrillation	1,780 (0.01)	1,235 (0.01)	545 (0.03)
Heart failure/arrest during surgery or procedure	70 (0.00)	NR[1]	NR[1]
Puerperal cerebrovascular disorders	4,515 (0.03)	2,775 (0.02)	1,740 (0.09)
Pulmonary edema/acute heart failure	12,180 (0.08)	5,935 (0.04)	6,244 (0.31)
Conversion of cardiac rhythm	1,545 (0.01)	1,090 (0.01)	455 (0.02)
Cardiogenic shock	4,370 (0.03)	3,310 (0.02)	1,060 (0.05)

[1]NR = Not reported as unweighted n ≤10.

© 2024 Malhamé I et al. *JAMA Network Open.*

References

1. Division of Reproductive Health National Center for Chronic Disease Prevention and Health Promotion. How Does CDC Identify Severe Maternal Morbidity? July 10, 2023. Accessed July 10, 2023. https://www.cdc.gov/reproductivehealth/maternalinfanthealth/smm/severe-morbidity-ICD.htm

© 2024 Malhamé I et al. *JAMA Network Open.*
