# Supplementary material for: Hypertensive Disorders and Cardiovascular Severe Maternal Morbidity in the US, 2015-2019
Source: JAMA Netw Open. 2024 Oct 3;7(10):e2436478. doi: 10.1001/jamanetworkopen.2024.36478 (PMC11581633; doi:10.1001/jamanetworkopen.2024.36478)
Supplement: Supplement 2. — Data Sharing Statement [file jamanetwopen-e2436478-s002.pdf]

Data Sharing Statement

Malhamé. Hypertensive Disorders and Cardiovascular Severe Maternal Morbidity in the US, 2015-2019. *JAMA Netw Open*. Published October 03, 2024.
doi:10.1001/jamanetworkopen.2024.36478

Data
Data available: No
